# COVID-19 impacts on the breast cancer care pathway among systemically marginalized communities in Ontario

**DOI:** 10.1007/s10552-025-02063-7

**Published:** 2025-09-24

**Authors:** Aisha K. Lofters, Priya Premranjith, Anastasia Gayowksy, Ielaf Khalil, Andrea M. Covelli, Juliet M. Daniel

**Affiliations:** 1 Peter Gilgan Centre for Women’s Cancers, Women’s College Hospital, Toronto, ON Canada; 2 Department of Family and Community Medicine, University of Toronto, Toronto, ON Canada; 3 ICES McMaster, Hamilton, ON Canada; 4 Lunenfeld-Tanenbaum Research Institute, Sinai Health System, Toronto, ON Canada; 5 Division of General Surgery, Department of Surgery, University of Toronto, Toronto, ON Canada; 6 Division of Surgical Oncology, Department of Surgery, Mount Sinai Hospital, Toronto, ON Canada; 7 Institute for Health Policy, Management, and Evaluation, University of Toronto, Toronto, ON Canada; 8 Department of Biology and Centre for Discovery in Cancer Research (CDCR), McMaster University, Hamilton, ON Canada

**Keywords:** Breast cancer, Cancer screening, COVID-19, Cancer care, Immigrant health

## Abstract

**Purpose:**

Healthcare system pauses occurred worldwide due to COVID-19, and may have worsened pre-existing disparities in breast cancer care. In this population-based, retrospective cohort study, we investigated indicators of breast cancer care (i.e., adherence to screening guidelines, early vs. late-stage diagnosis, and mastectomy vs. breast-conserving surgery) before and after COVID-19 lockdowns in Ontario, with an emphasis on immigrant women.

**Methods:**

We had three binary outcomes and corresponding cohorts, and each outcome/cohort was ascertained relative to two time periods: April 1, 2018-March 31, 2020 (“pre-pandemic”) and April 1, 2020-March 31, 2022 (“pandemic”): i) up to date on screening, ii) early vs late stage of breast cancer diagnosis, and iii) mastectomy vs breast-conserving surgery at any time after diagnosis for women who were diagnosed at stages I-III during each two-year time period. We conducted descriptive analyses, and used logistic regression, both unadjusted and adjusted, to determine odds ratios for our dichotomous outcomes.

**Results:**

Breast cancer screening rates dropped from 59.4% to 51.0%, and the number of women diagnosed dropped from 18,821 to 14,269, in the pre-pandemic vs pandemic period. In multivariable analyses, screening significantly dropped (AOR = 0.69 [95% CI (0.69–0.69)]), there was no significant difference for diagnostic stage (AOR = 0.99 [95% CI (0.92–1.05)]), and the use of mastectomy vs breast-conserving surgery was higher in the pandemic period (AOR = 1.14 [95% CI (1.08–1.20)]). Women from the Caribbean had lower odds of early-stage diagnosis in the pre-pandemic period despite a screening advantage.

**Conclusion:**

Future work should further explore the reasons for these findings and potential system-level solutions.

## Background

The COVID-19 pandemic substantially increased demands on healthcare services globally, with Intensive Care Units (ICU) capacity limits exceeded in many jurisdictions [1–3]. In Canada, as in many parts of the world, many healthcare services, such as breast cancer screening and some cancer treatments, including surgeries, were paused during the initial lockdowns to focus on COVID-19 management and to adhere to public health restrictions [4, 5]. While necessary at the time, the effects of these healthcare system pauses are still being felt; in March 2022, the proportion of people up to date on breast cancer screening in Ontario (Canada’s most populous province) was only 51.7% as compared to 61.1% in March 2019 [6]. During the first pandemic year, cancer treatment surgical procedures were down by 14.1% and radiation treatment visits were down by 21.0% [7]. Although there had been shifts towards breast-conserving surgery instead of mastectomy for early-stage breast cancers over time [8], as in other jurisdictions, mastectomies accounted for a higher proportion of breast cancer surgeries in Ontario during the pandemic [9–11].

Many systemically marginalized communities, such as foreign-born Canadians and those living with low income, were disproportionately impacted by COVID-19 across Canada, including in the province of Ontario [12]. These same groups have historically been subject to breast cancer care disparities. Immigrants in Ontario are less likely to be up to date on screening for breast cancer, with Vahabi et al*.* finding 57% of identified immigrants being up to date versus 66% for long-term residents [13–15]. In Ontario, women who immigrated from Latin America, the Caribbean and South Asia were found to be less likely to have a screen-detected cancer, to have a longer median diagnostic interval, and to be diagnosed at a later stage than long-term Ontario residents [16]. For example, 37.9% of long-term Ontario residents were diagnosed at stage I in a 2019 study versus 26.2% of women from Latin America and the Caribbean [16]. Women of South Asian ancestry were found to be diagnosed with breast cancer at a later stage than other women [17], and patients with higher socioeconomic status were found to be more likely to be diagnosed with breast cancer at an earlier stage and to have better overall survival [18].

The lockdowns and healthcare system pauses that occurred due to COVID-19 may have worsened these pre-existing disparities in breast cancer care. In this population-based, retrospective cohort study, we aimed to investigate indicators of breast cancer care (i.e., adherence to screening guidelines, early vs. late-stage diagnosis, and mastectomy vs. breast-conserving surgery) before and after COVID-19 lockdowns in Ontario, with an emphasis on women who are systemically marginalized.

## Methods

### Study setting

Ontario is Canada’s largest population by province: according to the 2021 Canadian Census, 38.5% of Canadians live in Ontario [19]. Ontario also has a sizeable immigrant population, with 30% of residents born outside of Canada [19]. Ontario, like other Canadian provinces, has a universal healthcare system where medically necessary healthcare services, including cancer screening, diagnosis and treatment, are covered for all Canadian citizens and permanent residents who live in the province.

### Data sources

All data were obtained from ICES, which is an independent, non-profit research institute funded by an annual grant from the Ontario Ministry of Health (MOH) and the Ministry of Long-Term Care (MLTC). As a prescribed entity under Ontario’s privacy legislation, ICES is authorized to collect and use health care data for the purposes of health system analysis, evaluation and decision support. Secure access to these data is governed by policies and procedures that are approved by the Information and Privacy Commissioner of Ontario.

We accessed several datasets for this study (see Box 1), including the Ontario Cancer Registry (OCR) which is the provincial database of information about all Ontario residents diagnosed with cancer, and the Immigration, Refugee & Citizenship Canada Permanent Resident (IRCC-PR) database which was used to ascertain immigrant status and contains information on all permanent residents since 1985, including country of birth. The Registered Persons Database (RPDB) contains demographic information on persons registered under the province’s universal Ontario Health Insurance Plan (OHIP) and the Census was used to determine neighbourhood characteristics based on cohort member’s postal codes of residence. The datasets were linked using unique encoded identifiers and analyzed at ICES.

Box 1. Datasets used in this study

**Table:** 

Ontario Breast Screening Program (OBSP)	Includes key data elements of breast cancer screening encounters that occur through the provincial screening program
Ontario Cancer Registry (OCR)	Provincial database of information about all Ontario residents diagnosed with incident cancers
Ontario Health Insurance Plan (OHIP)	Includes physician visits, diagnostic codes, and billing codes for physicians
Discharge Abstract Database (DAD)	Contains records of inpatient hospitalizations including diagnoses and procedures
National Ambulatory Care Reporting System (NACRS)	Contains records of emergency department and outpatient cancer care clinics
Registered Persons Database (RPDB)	Includes each individual’s health card number, date of birth, sex (gender not available), and postal code
Immigration, Refugee & Citizenship Canada Permanent Resident (IRCC-PR)	Records information on all permanent residents to Ontario since 1985, including country of birth and immigration category
Ontario Marginalization Index	A data tool that combines a wide range of demographic indicators from the Census into four distinct dimensions of marginalization, including “racialized and newcomer populations”
Client Agency Program Enrolment (CAPE)	Contains patients’ registration information with a primary care organization
Cancer Activity Level Reporting (ALR)	Main database on systemic therapy for cancer care in Ontario

### Outcomes

We had three binary outcomes and each outcome was ascertained relative to two time periods: April 1, 2018-March 31, 2020 (considered “pre-pandemic”) and April 1, 2020-March 31, 2022 (considered “pandemic”): i) females up to date on screening based on provincial average-risk screening guidelines – i.e. at least one mammogram in each two-year time period for women who were screen-eligible as defined by an OBSP record or relevant OHIP billing codes, ii) early (stage I/II) vs late (stage III/V) stage of breast cancer diagnosis in the OCR during each two-year time period, and iii) mastectomy vs breast-conserving surgery, using NACRS, DAD and OHIP to identify surgical cases, at any time after diagnosis for women who were diagnosed at stages I-III during each two-year time period.

### Study population

We defined six cohorts of female residents of Ontario with a valid OHIP card, to correspond to our three outcomes and two time periods. For screening, we included those who were 52–69 years of age on the last day of each time period (March 31, 2020 and March 31, 2022), and thus age-eligible for screening during the entire time period, with no prior diagnosis of breast cancer and no prior mastectomy. Average risk screening eligibility in Ontario started at age 50 during our study time frame. Provincial guidelines recommend a mammogram every two years if results are normal [20]. For diagnostic stage, we included those with an incident case of breast cancer in the OCR during the time periods. For surgery type, we included those who had been diagnosed with stage I-III breast cancer at any time and had mastectomy or breast-conserving surgery during the two time periods. Cohort members also had to be eligible for OHIP at least three years prior to the index dates to ensure we could track relevant healthcare usage.

### Study variables

For cohort members, we also determined their immigrant status, and if they were an immigrant, their world region of origin and years since arrival were noted. At the neighborhood level, we used Census data to determine income quintile, the quintile of racialized and newcomer populations, whether the female lived in a rural area, and whether she had a primary care provider (PCP), either one she was formally enrolled with (known as “rostered”), or one not she was not formally enrolled with but had visited multiple times previously (known as “virtually rostered”).

### Analysis

We conducted descriptive analyses of the six cohorts for the two time periods, using standardized differences for comparison. We considered any standardized difference greater than 0.10 to be important [21]. We used logistic regression, both unadjusted and adjusted, to determine odds ratios for our dichotomous outcomes by our study variables both pre- and post- pandemic periods and overall. In adjusted analyses, our models included age, pre- vs. post-pandemic time periods (for the overall model), immigrant status and region of birth, income quintile and rural status, and primary care provider access as determined by rostering status. As people missing a value for residence may be unhoused or underhoused, and thus may be a particularly vulnerable group, they were included in analyses.

## Results

Table 1 depicts creation of the six cohorts, and baseline characteristics of the six cohorts are shown in Tables 2a–c. Screening rates dropped significantly between the two time periods (standardized difference 0.17). Approximately 12% of those eligible for screening lived in rural areas and approximately 20% were immigrants (Table 2a), who had been in Canada for a median of ~ 20 years, and for whom Asia was the most common country of origin. For women diagnosed with breast cancer (Table 2b), the absolute number of women dropped from 18,821 in the pre-pandemic period to 14,269 in the pandemic period. Of those diagnosed, 13.1% were diagnosed at stage III/IV, and the median age of diagnosis was 63 years; this did not significantly differ across the two time periods (Table 2b). For surgical outcomes (Table 2c), 26.6% vs 29.2% of women had a mastectomy across the time periods but this difference was not significant (SD = 0.058).

**Table 1:** Cohort creation

Screening Cohort	Pre-Pandemic (April 1, 2018—March 31, 2020)	Pandemic (April 1, 2020—March 31, 2022)
Females who were 52–69 years of age during the time periods with no prior diagnosis of breast cancer and no prior mastectomy	2,048,544	2,111,876
Non-Ontario resident at index date	103,014	114,257
Not OHIP eligible in 3 years preceding index date	261,222	292,669
Landing date greater than index date	260	111
INCLUDED	1,684,048	1,704,839
Diagnosis Cohort	Pre-Pandemic (April 1, 2018—March 31, 2020)	Pandemic (April 1, 2020—March 31, 2022)
Incident breast cancer diagnosis among women aged 18—105	20,896	20,809
Non-Ontario resident at index date or landing date after index date	125	92
Not OHIP eligible in 3 years preceding index date	573	599
Missing stage	1,377	5,848
INCLUDED	18,821	14,270
Surgery Cohort	Pre-Pandemic (April 1, 2018—March 31, 2020)	Pandemic (April 1, 2020—March 31, 2022)
Incident breast cancer diagnosis among women aged 18—105	16,661	16,304
Non-Ontario resident at index date or not OHIP eligible in 3 years preceding index date or landing date	405	358
INCLUDED	16,256	15,946

**Table 2:** .

a.Baseline characteristics of breast cancer screening cohorts					
Variable	Value	Total	Pre-Pandemic (April 1, 2018—March 31, 2020)	Pandemic (April 1, 2020—March 31, 2022)	Standardized difference
Time period	Sample Size	*n* = 3,388,308	*n* = 1,683,780	*n* = 1,704,528	
Up to date on screening	No—*n* (%)	1,519,775 (44.9%)	683,950 (40.6%)	835,825 (49.0%)	0.17
	Yes—*n* (%)	1,868,533 (55.1%)	999,830 (59.4%)	868,703 (51.0%)	0.17
Age	Mean (SD)	59.86 (5.06)	59.76 (5.09)	59.96 (5.04)	0.04
	Median (Q1-Q3)	60.00 (56.00–64.00)	59.00 (55.00–64.00)	60.00 (56.00–64.00)	0.042
	Missing Data (%)	0.0%	0.0%	0.0%	
Income quintile and rural status	Missing—*n* (%)	6,528 (0.2%)	4,068 (0.2%)	2,460 (0.1%)	0.022
	Q1—*n* (%)	567,422 (16.7%)	277,728 (16.5%)	289,694 (17.0%)	0.013
	Q2—*n* (%)	593,339 (17.5%)	293,191 (17.4%)	300,148 (17.6%)	0.005
	Q3—*n* (%)	586,426 (17.3%)	294,441 (17.5%)	291,985 (17.1%)	0.009
	Q4—*n* (%)	589,816 (17.4%)	292,595 (17.4%)	297,221 (17.4%)	0.002
	Q5—*n* (%)	642,295 (19.0%)	319,935 (19.0%)	322,360 (18.9%)	0.002
	Rural—*n* (%)	402,482 (11.9%)	201,822 (12.0%)	200,660 (11.8%)	0.007
Racialized and Newcomer Populations Quintile	Missing—*n* (%)	26,037 (0.8%)	14,366 (0.9%)	11,671 (0.7%)	0.019
	Q1 (lowest)—*n* (%)	623,355 (18.4%)	314,660 (18.7%)	308,695 (18.1%)	0.015
	Q2—*n* (%)	620,417 (18.3%)	306,448 (18.2%)	313,969 (18.4%)	0.006
	Q3—*n* (%)	615,610 (18.2%)	306,267 (18.2%)	309,343 (18.1%)	0.001
	Q4—*n* (%)	673,062 (19.9%)	329,199 (19.6%)	343,863 (20.2%)	0.016
	Q5 (highest)—*n* (%)	829,827 (24.5%)	412,840 (24.5%)	416,987 (24.5%)	0.001
Rostering status	Not rostered—*n* (%)	240,698 (7.1%)	123,734 (7.3%)	116,964 (6.9%)	0.019
	Rostered—*n* (%)	2,770,858 (81.8%)	1,374,529 (81.6%)	1,396,329 (81.9%)	0.007
	Virtually rostered—*n* (%)	376,752 (11.1%)	185,517 (11.0%)	191,235 (11.2%)	0.006
Immigrant status	No—*n* (%)	2,723,916 (80.4%)	1,368,295 (81.3%)	1,355,621 (79.5%)	0.044
	Yes—*n* (%)	664,392 (19.6%)	315,485 (18.7%)	348,907 (20.5%)	0.044
Years since landing	Mean (SD)	20.45 (7.92)	19.73 (7.70)	21.10 (8.05)	0.174
	Median (Q1-Q3)	21.54 (14.65–26.85)	20.91 (13.95–25.89)	22.28 (15.14–27.59)	0.18
	Missing Data (%)	80.4%	81.3%	79.5%	
Region of birth	Asia—*n* (%)	344,386 (10.2%)	163,743 (9.7%)	180,643 (10.6%)	0.029
	Australia/North America—*n* (%)	27,482 (0.8%)	13,303 (0.8%)	14,179 (0.8%)	0.005
	Caribbean—*n* (%)	45,133 (1.3%)	21,331 (1.3%)	23,802 (1.4%)	0.011
	Europe/Former USSR—*n* (%)	126,971 (3.7%)	60,882 (3.6%)	66,089 (3.9%)	0.014
	North Africa/Middle East—*n* (%)	52,107 (1.5%)	24,116 (1.4%)	27,991 (1.6%)	0.017
	Not an immigrant—*n* (%)	2,723,916 (80.4%)	1,368,295 (81.3%)	1,355,621 (79.5%)	0.044
	South America—*n* (%)	36,837 (1.1%)	17,626 (1.0%)	19,211 (1.1%)	0.008
	Sub-Saharan Africa—*n* (%)	31,476 (0.9%)	14,484 (0.9%)	16,992 (1.0%)	0.014

b. Baseline characteristics of breast cancer diagnosis cohorts					
Time period	Sample Size	*n* = 33,090	*n* = 18,821	*n* = 14,269	
Cancer stage	0—*n* (%)	258 (0.8%)	70 (0.4%)	188 (1.3%)	0.103
	I/II—*n* (%)	28,506 (86.1%)	16,305 (86.6%)	12,201 (85.5%)	0.032
	III/IV—*n* (%)	4,326 (13.1%)	2,446 (13.0%)	1,880 (13.2%)	0.005
Age	Mean (SD)	62.55 (13.27)	62.74 (13.32)	62.31 (13.20)	0.032
	Median (Q1-Q3)	63.00 (53.00–72.00)	63.00 (53.00–72.00)	63.00 (53.00–72.00)	0.025
	Missing Data (%)	0.0%	0.0%	0.0%	
Income quintile and rural status	Missing—*n* (%)	49 (0.1%)	29 (0.2%)	20 (0.1%)	0.004
	Q1—*n* (%)	5,563 (16.8%)	3,156 (16.8%)	2,407 (16.9%)	0.003
	Q2—*n* (%)	5,784 (17.5%)	3,347 (17.8%)	2,437 (17.1%)	0.019
	Q3—*n* (%)	5,574 (16.8%)	3,174 (16.9%)	2,400 (16.8%)	0.001
	Q4—*n* (%)	5,908 (17.9%)	3,361 (17.9%)	2,547 (17.8%)	0
	Q5—*n* (%)	6,530 (19.7%)	3,686 (19.6%)	2,844 (19.9%)	0.009
	Rural—*n* (%)	3,682 (11.1%)	2,068 (11.0%)	1,614 (11.3%)	0.01
	Q5 (highest)—*n* (%)	8,135 (24.6%)	4,658 (24.7%)	3,477 (24.4%)	0.009
Racialized and Newcomer Populations Quintile	Missing—*n* (%)	207 (0.6%)	108 (0.6%)	99 (0.7%)	0.015
	Q1 (lowest)—*n* (%)	6,029 (18.2%)	3,440 (18.3%)	2,589 (18.1%)	0.003
	Q2—*n* (%)	6,448 (19.5%)	3,640 (19.3%)	2,808 (19.7%)	0.009
	Q3—*n* (%)	6,499 (19.6%)	3,588 (19.1%)	2,911 (20.4%)	0.034
	Q4—*n* (%)	6,694 (20.2%)	3,809 (20.2%)	2,885 (20.2%)	0
	Q5 (highest)—*n* (%)	7,213 (21.8%)	4,236 (22.5%)	2,977 (20.9%)	0.04
Rostering status	Not rostered—*n* (%)	744 (2.2%)	419 (2.2%)	325 (2.3%)	0.003
	Rostered—*n* (%)	28,397 (85.8%)	16,211 (86.1%)	12,186 (85.4%)	0.021
	Virtually rostered—*n* (%)	3,949 (11.9%)	2,191 (11.6%)	1,758 (12.3%)	0.021
Immigrant status	No—*n* (%)	27,618 (83.5%)	15,739 (83.6%)	11,879 (83.3%)	0.01
	Yes—*n* (%)	5,472 (16.5%)	3,082 (16.4%)	2,390 (16.7%)	0.01
Years since landing	Mean (SD)	19.66 (8.49)	19.17 (8.31)	20.29 (8.67)	0.132
	Median (Q1-Q3)	20.00 (12.92–26.74)	19.57 (12.61–26.23)	20.39 (13.49–27.65)	0.129
	Missing Data (%)	83.5%	83.6%	83.3%	
Region of birth	Asia—*n* (%)	2,723 (8.2%)	1,545 (8.2%)	1,178 (8.3%)	0.002
	Australia/North America—*n* (%)	230 (0.7%)	129 (0.7%)	101 (0.7%)	0.003
	Caribbean—*n* (%)	358 (1.1%)	205 (1.1%)	153 (1.1%)	0.002
	Europe/Former USSR—*n* (%)	1,079 (3.3%)	599 (3.2%)	480 (3.4%)	0.01
	North Africa/Middle East—*n* (%)	621 (1.9%)	343 (1.8%)	278 (1.9%)	0.009
	Not an immigrant—*n* (%)	27,618 (83.5%)	15,739 (83.6%)	11,879 (83.3%)	0.01
	South America—*n* (%)	245 (0.7%)	142 (0.8%)	103 (0.7%)	0.004
	Sub-Saharan Africa—*n* (%)	216 (0.7%)	119 (0.6%)	97 (0.7%)	0.006

c. Baseline characteristics of surgery cohorts					
Time period	Sample Size	*n* = 32,201	*n* = 16,256	*n* = 15,945	
Type of Surgery	Breast conserving—*n* (%)	23,237 (72.2%)	11,940 (73.4%)	11,297 (70.8%)	0.058
	Mastectomy—*n* (%)	8,964 (27.8%)	4,316 (26.6%)	4,648 (29.2%)	0.058
Age	Mean (SD)	62.44 (12.81)	62.36 (12.81)	62.53 (12.82)	0.013
	Median (Q1-Q3)	63.00 (53.00–72.00)	63.00 (53.00–72.00)	63.00 (53.00–72.00)	0.015
	Missing Data (%)	0.0%	0.0%	0.0%	
Income quintile and rural status	Missing—*n* (%)	50 (0.2%)	25 (0.2%)	25 (0.2%)	0.001
	Q1—*n* (%)	5,209 (16.2%)	2,619 (16.1%)	2,590 (16.2%)	0.004
	Q2—*n* (%)	5,580 (17.3%)	2,852 (17.5%)	2,728 (17.1%)	0.012
	Q3—*n* (%)	5,502 (17.1%)	2,726 (16.8%)	2,776 (17.4%)	0.017
	Q4—*n* (%)	5,861 (18.2%)	2,963 (18.2%)	2,898 (18.2%)	0.001
	Q5—*n* (%)	6,399 (19.9%)	3,238 (19.9%)	3,161 (19.8%)	0.002
	Rural—*n* (%)	3,600 (11.2%)	1,833 (11.3%)	1,767 (11.1%)	0.006
Racialized and Newcomer Populations Quintile	Missing—*n* (%)	196 (0.6%)	88 (0.5%)	108 (0.7%)	0.017
	Q1 (lowest)—*n* (%)	5,787 (18.0%)	2,979 (18.3%)	2,808 (17.6%)	0.019
	Q2—*n* (%)	6,180 (19.2%)	3,161 (19.4%)	3,019 (18.9%)	0.013
	Q3—*n* (%)	6,154 (19.1%)	3,078 (18.9%)	3,076 (19.3%)	0.009
	Q4—*n* (%)	6,531 (20.3%)	3,288 (20.2%)	3,243 (20.3%)	0.003
	Q5 (highest)—*n* (%)	7,353 (22.8%)	3,662 (22.5%)	3,691 (23.1%)	0.015
Rostering status	Not rostered—*n* (%)	511 (1.6%)	257 (1.6%)	254 (1.6%)	0.001
	Rostered—*n* (%)	27,941 (86.8%)	14,160 (87.1%)	13,781 (86.4%)	0.02
	Virtually rostered—*n* (%)	3,749 (11.6%)	1,839 (11.3%)	1,910 (12.0%)	0.021
Immigrant status	No—*n* (%)	26,704 (82.9%)	13,580 (83.5%)	13,124 (82.3%)	0.033
	Yes—*n* (%)	5,497 (17.1%)	2,676 (16.5%)	2,821 (17.7%)	0.033
Years since landing	Mean (SD)	19.89 (8.53)	19.28 (8.39)	20.47 (8.62)	0.139
	Median (Q1-Q3)	20.33 (13.30–27.00)	19.68 (12.82–26.34)	20.68 (13.85–27.76)	0.136
	Missing Data (%)	82.9%	83.5%	82.3%	
Region of birth	Asia—*n* (%)	2,781 (8.6%)	1,333 (8.2%)	1,448 (9.1%)	0.031
	Australia/North America—*n* (%)	221 (0.7%)	120 (0.7%)	101 (0.6%)	0.013
	Caribbean—*n* (%)	380 (1.2%)	187 (1.2%)	193 (1.2%)	0.006
	Europe/Former USSR—*n* (%)	1,039 (3.2%)	520 (3.2%)	519 (3.3%)	0.003
	North Africa/Middle East—*n* (%)	611 (1.9%)	291 (1.8%)	320 (2.0%)	0.016
	Not an immigrant—*n* (%)	26,704 (82.9%)	13,580 (83.5%)	13,124 (82.3%)	0.033
	South America—*n* (%)	253 (0.8%)	128 (0.8%)	125 (0.8%)	0
	Sub-Saharan Africa—*n* (%)	212 (0.7%)	97 (0.6%)	115 (0.7%)	0.015

### Unadjusted analyses

#### Screening

In unadjusted analyses, there was an income gradient seen for screening, with the lowest vs. highest neighbourhood income quintile having an odds ratio of 0.57 (95% CI [0.57–0.58]) overall and for both time periods (Table 3a). Rural residents were also less likely to be screened than those in the highest-income neighbourhoods (overall OR = 0.82 (95% CI [0.81–0.83]). Differences in screening were also seen by racialized and newcomer populations quintile; these differences grew in the pandemic period (highest vs lowest quintile OR = 0.83 (95% CI [0.82–0.84]) in pre-pandemic period; OR = 0.77 (95% CI [0.76–0.78] in pandemic period). People who had no primary care had the most dramatic differences in screening, although the differences were smaller in the pandemic period (not rostered vs. rostered OR = 0.10 (95% CI [0.10–0.10] in pre-pandemic period vs. OR = 0.14 (95% CI [0.14–0.14]) in pandemic period). Immigrants from Europe and the former USSR countries had the lowest odds of screening relative to non-immigrants: OR = 0.62 [95% CI (0.61–0.63) while those from the Caribbean had the highest: OR = 1.00 [95% CI (0.98–1.02)]. Odds of screening relative to non-immigrants dropped most noticeably for women from Asia: OR = 0.74 [95% CI (0.73–0.75] pre-pandemic vs. OR = 0.66 [95% CI (0.65–0.67)] in the pandemic period.

**Table 3:** .

a.Unadjusted odds ratios for breast cancer screening							
		Overall		Pre-pandemic (April 1, 2018—March 31, 2020)		Pandemic (April 1, 2020—March 31, 2022)	
Variable	Effect	Odds ratio	95% confidence interval	Odds ratio	95% confidence interval	Odds ratio	95% confidence interval
Time period	Pandemic (April 1, 2020—March 31, 2022) vs Pre-pandemic (April 1, 2018—March 31, 2020)	0.71	(0.71, 0.71)				
Age		1.02	(1.02, 1.02)	1.02	(1.02, 1.02)	1.02	(1.01, 1.02)
Income quintile and rural status	Missing vs Q5 (highest)	0.61	(0.58, 0.64)	0.61	(0.57, 0.64)	0.55	(0.50, 0.59)
	Q1 (lowest) vs Q5	0.57	(0.57, 0.58)	0.57	(0.57, 0.58)	0.57	(0.57, 0.58)
	Q2 vs Q5	0.72	(0.71, 0.72)	0.73	(0.72, 0.73)	0.71	(0.70, 0.72)
	Q3 vs Q5	0.82	(0.82, 0.83)	0.83	(0.82, 0.84)	0.81	(0.80, 0.82)
	Q4 vs Q5	0.90	(0.90, 0.91)	0.92	(0.91, 0.93)	0.89	(0.88, 0.90)
	Rural vs Q5	0.82	(0.81, 0.83)	0.82	(0.81, 0.83)	0.82	(0.81, 0.83)
Racialized and Newcomer Populations Quintile	Missing vs Q1 (lowest)	0.72	(0.70, 0.74)	0.72	(0.69, 0.74)	0.70	(0.67, 0.72)
	Q2 vs Q1 (lowest)	1.03	(1.02, 1.04)	1.01	(1.00, 1.02)	1.05	(1.04, 1.06)
	Q3 vs Q1 (lowest)	1.00	(1.00, 1.01)	1.00	(0.99, 1.01)	1.01	(1.00, 1.02)
	Q4 vs Q1 (lowest)	0.93	(0.92, 0.93)	0.94	(0.93, 0.95)	0.92	(0.91, 0.93)
	Q5 (highest) vs Q1 (lowest)	0.80	(0.79, 0.80)	0.83	(0.82, 0.84)	0.77	(0.77, 0.78)
Rostering status	Not rostered vs Rostered	0.12	(0.12, 0.12)	0.10	(0.10, 0.10)	0.14	(0.14, 0.14)
	Virtually rostered vs Rostered	0.61	(0.61, 0.62)	0.60	(0.60, 0.61)	0.62	(0.61, 0.62)
Region of birth	Asia vs Not an immigrant	0.69	(0.69, 0.70)	0.74	(0.73, 0.75)	0.66	(0.65, 0.67)
	Australia/North America vs Not an immigrant	0.69	(0.68, 0.71)	0.67	(0.65, 0.70)	0.72	(0.70, 0.75)
	Caribbean vs Not an immigrant	1.00	(0.98, 1.02)	1.03	(1.00, 1.06)	1.00	(0.97, 1.03)
	Europe/Former USSR vs Not an immigrant	0.62	(0.61, 0.63)	0.61	(0.60, 0.62)	0.63	(0.62, 0.64)
	North Africa/Middle East vs Not an immigrant	0.73	(0.72, 0.74)	0.74	(0.72, 0.76)	0.74	(0.72, 0.76)
	South America vs Not an immigrant	0.99	(0.97, 1.01)	1.00	(0.97, 1.03)	0.99	(0.96, 1.02)
	Sub-Saharan Africa vs Not an immigrant	0.65	(0.63, 0.66)	0.67	(0.64, 0.69)	0.64	(0.62, 0.66)

b. Unadjusted odds ratios for early vs late state of diagnosis							
Time Period	Pandemic (April 1, 2020—March 31, 2022) vs Pre-pandemic (April 1, 2018—March 31, 2020)	0.98	(0.92, 1.05)				
Age		1.00	(1.00, 1.00)	1.00	(0.99, 1.00)	1.00	(1.00, 1.01)
Income quintile and rural status	Missing vs Q5 (highest)	0.39	(0.20, 0.75)	0.49	(0.20, 1.20)	0.29	(0.11, 0.76)
	Q1 (lowest) vs Q5	0.72	(0.65, 0.80)	0.71	(0.62, 0.82)	0.73	(0.62, 0.86)
	Q2 vs Q5	0.79	(0.71, 0.88)	0.81	(0.70, 0.93)	0.78	(0.66, 0.92)
	Q3 vs Q5	0.79	(0.71, 0.88)	0.80	(0.69, 0.92)	0.78	(0.66, 0.92)
	Q4 vs Q5	0.86	(0.77, 0.95)	0.88	(0.76, 1.02)	0.82	(0.70, 0.97)
	Rural vs Q5	0.92	(0.82, 1.05)	1.02	(0.86, 1.21)	0.83	(0.68, 0.99)
Racialized and Newcomer Populations Quintile	Missing vs Q1 (lowest)	0.62	(0.43, 0.88)	0.73	(0.44, 1.22)	0.53	(0.33, 0.86)
	Q2 vs Q1 (lowest)	1.04	(0.93, 1.15)	1.00	(0.87, 1.15)	1.09	(0.93, 1.28)
	Q3 vs Q1 (lowest)	1.02	(0.92, 1.13)	1.07	(0.93, 1.24)	0.96	(0.82, 1.13)
	Q4 vs Q1 (lowest)	0.98	(0.88, 1.09)	0.95	(0.83, 1.09)	1.03	(0.88, 1.20)
	Q5 (highest) vs Q1 (lowest)	0.92	(0.83, 1.02)	0.90	(0.79, 1.03)	0.95	(0.81, 1.10)
Rostering status	Not rostered vs Rostered	0.38	(0.32, 0.45)	0.37	(0.30, 0.47)	0.39	(0.30, 0.50)
	Virtually rostered vs Rostered	0.69	(0.63, 0.75)	0.68	(0.60, 0.77)	0.70	(0.61, 0.80)
Region of birth	Asia vs Not an immigrant	0.97	(0.86, 1.09)	0.92	(0.79, 1.07)	1.03	(0.86, 1.23)
	Australia/North America vs Not an immigrant	1.22	(0.81, 1.86)	1.31	(0.74, 2.34)	1.13	(0.61, 2.06)
	Caribbean vs Not an immigrant	0.71	(0.54, 0.94)	0.63	(0.44, 0.89)	0.86	(0.55, 1.34)
	Europe/Former USSR vs Not an immigrant	0.93	(0.78, 1.11)	1.06	(0.83, 1.36)	0.81	(0.63, 1.04)
	North Africa/Middle East vs Not an immigrant	1.09	(0.85, 1.39)	0.95	(0.69, 1.30)	1.30	(0.88, 1.92)
	South America vs Not an immigrant	1.44	(0.94, 2.22)	1.25	(0.73, 2.13)	1.80	(0.87, 3.71)
	Sub-Saharan Africa vs Not an immigrant	0.60	(0.43, 0.84)	0.50	(0.33, 0.77)	0.77	(0.45, 1.32)

c. Unadjusted odds ratios for mastectomy vs breast-conserving surgery							
Time Period	Pandemic (April 1, 2020—March 31, 2022) vs Pre-pandemic (April 1, 2018—March 31, 2020)	1.14	(1.08, 1.20)				
Age		0.99	(0.99, 0.99)	0.99	(0.98, 0.99)	0.99	(0.99, 0.99)
Income quintile and rural status	Missing vs Q5	1.55	(0.87, 2.77)	1.18	(0.49, 2.82)	1.98	(0.89, 4.37)
	Q1 vs Q5	1.11	(1.03, 1.21)	1.09	(0.97, 1.23)	1.13	(1.01, 1.27)
	Q2 vs Q5	1.10	(1.01, 1.19)	1.18	(1.05, 1.32)	1.03	(0.92, 1.15)
	Q3 vs Q5	1.03	(0.95, 1.12)	1.01	(0.90, 1.14)	1.04	(0.93, 1.17)
	Q4 vs Q5	1.11	(1.03, 1.20)	1.18	(1.06, 1.33)	1.05	(0.94, 1.17)
	Rural vs Q5	1.01	(0.93, 1.11)	1.11	(0.97, 1.26)	0.93	(0.82, 1.06)
Racialized and Newcomer Populations Quintile	Missing vs Q1 (lowest)	1.60	(1.19, 2.15)	1.20	(0.75, 1.91)	1.96	(1.33, 2.89)
	Q2 vs Q1 (lowest)	1.10	(1.02, 1.19)	1.13	(1.01, 1.27)	1.07	(0.95, 1.20)
	Q3 vs Q1 (lowest)	1.00	(0.93, 1.09)	0.96	(0.85, 1.08)	1.04	(0.93, 1.17)
	Q4 vs Q1 (lowest)	0.97	(0.90, 1.05)	0.95	(0.85, 1.07)	0.99	(0.88, 1.10)
	Q5 (highest) vs Q1 (lowest)	1.11	(1.02, 1.19)	1.11	(1.00, 1.24)	1.10	(0.98, 1.22)
Rostering status	Not rostered vs Rostered	1.06	(0.87, 1.28)	0.98	(0.74, 1.29)	1.14	(0.87, 1.48)
	Virtually rostered vs Rostered	1.16	(1.07, 1.25)	1.19	(1.07, 1.32)	1.12	(1.01, 1.25)
Region of birth	Asia vs Not an immigrant	1.42	(1.31, 1.55)	1.44	(1.27, 1.62)	1.41	(1.25, 1.57)
	Australia/North America vs Not an immigrant	1.33	(1.00, 1.76)	1.39	(0.95, 2.04)	1.27	(0.84, 1.93)
	Caribbean vs Not an immigrant	0.88	(0.70, 1.12)	1.11	(0.80, 1.53)	0.70	(0.49, 0.98)
	Europe/Former USSR vs Not an immigrant	1.13	(0.98, 1.29)	1.15	(0.94, 1.39)	1.11	(0.92, 1.34)
	North Africa/Middle East vs Not an immigrant	1.11	(0.93, 1.32)	1.23	(0.95, 1.58)	1.00	(0.78, 1.27)
	South America vs Not an immigrant	0.80	(0.60, 1.07)	0.77	(0.50, 1.18)	0.83	(0.55, 1.24)
	Sub-Saharan Africa vs Not an immigrant	0.85	(0.62, 1.17)	0.85	(0.52, 1.36)	0.85	(0.55, 1.29)

#### Diagnosis

An income gradient was again seen in both time periods for early vs late diagnosis (Table 3b) and those who were not rostered to a primary care provider had much lower odds of early diagnosis in unadjusted analyses: OR = 0.38 [95% CI (0.32–0.45)]. In the pre-pandemic period, women from the Caribbean and Sub-Saharan Africa had significantly lower odds of early-stage diagnosis; OR = 0.63 [95% CI (0.44–0.89)] and OR = 0.50 [95% CI (0.33–0.77)] respectively. Results were not significant in the pandemic period. In the pandemic period, rural residents were less likely to be diagnosed early (OR = 0.83 [95% CI (0.68–0.99)].

#### Surgery

As described in Table 3c, those in the lowest vs. highest income quintile were significantly more likely in unadjusted analyses to have a mastectomy overall and in both time periods (overall OR = 1.11 [95% CI (1.03–1.21)]), as were those who were virtually rostered vs formally rostered (overall OR = 1.16 [95% CI (1.07–1.25)]. Women from Asia had 1.42 [95% CI (1.31–1.55]) times higher odds of mastectomy vs non-immigrants, with little change between time periods. Women from the Caribbean had the most notable change between time periods, with a pre-pandemic odds ratio of 1.11 [95% CI (0.80–1.53)] vs 0.70 [95% CI (0.49–0.98)] afterward; however, confidence intervals are overlapping.

### Multivariable analyses

#### Screening

For each time period and overall, we conducted multivariable logistic regression for each outcome, adjusting for age, income quintile, rostering status, and region of birth. In the overall model for screening (Table 4a), the adjusted odds ratio (AOR) of screening in the pandemic period were 0.69 [95% CI (0.69–0.69)] times those in the pre-pandemic period. A clear income gradient was still seen overall and in both time periods in the model, and rural residents had an overall AOR of 0.83 (95% CI [0.82–0.84]). Those who were not rostered had the lowest adjusted relative odds (overall AOR = 0.12 [95% CI (0.12–0.12)]. After adjustment, women from the Caribbean had the highest relative odds of screening in both time periods (AOR = 1.19 [95% CI (1.16–1.23) and 1.14 [95% CI (1.11–1.17)] respectively) and women from Europe, the former USSR and Sub-Saharan Africa had the lowest.

**Table 4:** .

a.Multivariable logistic regression for breast cancer screening, adjusting for age, income quintile, rostering status, and region of birth							
		Overall		Pre-pandemic (April 1, 2018—March 31, 2020)		Pandemic (April 1, 2020—March 31, 2022)	
Variable	Effect	Odds ratio	95% confidence interval	Odds ratio	95% confidence interval	Odds ratio	95% confidence interval
Region of birth	Asia vs not an immigrant	0.79	(0.78, 0.79)	0.86	(0.85, 0.87)	0.73	(0.72, 0.74)
	Australia/North America vs not an immigrant	0.85	(0.83, 0.87)	0.86	(0.82, 0.89)	0.85	(0.82, 0.87)
	Caribbean vs not an immigrant	1.16	(1.14, 1.18)	1.19	(1.16, 1.23)	1.14	(1.11, 1.17)
	Europe/Former USSR vs Not an immigrant	0.69	(0.68, 0.70)	0.69	(0.68, 0.70)	0.69	(0.68, 0.70)
	North Africa/Middle East vs Not an immigrant	0.87	(0.85, 0.88)	0.89	(0.87, 0.92)	0.84	(0.82, 0.86)
	South America vs not an immigrant	1.12	(1.10, 1.15)	1.15	(1.11, 1.18)	1.10	(1.07, 1.14)
	Sub-Saharan Africa vs not an immigrant	0.79	(0.77, 0.80)	0.82	(0.79, 0.85)	0.76	(0.74, 0.78)
Time Period	Pandemic (April 1, 2020—March 31, 2022) vs Pre-pandemic (April 1, 2018—March 31, 2020)	0.69	(0.69, 0.69)	1.02	(1.02, 1.02)	1.01	(1.01, 1.01)
Age		1.02	(1.01, 1.02)	0.67	(0.63, 0.72)	0.63	(0.58, 0.68)
Income quintile and rural status	Missing vs Q5	0.65	(0.62, 0.69)				
	Q1 vs Q5	0.62	(0.61, 0.62)	0.62	(0.61, 0.63)	0.62	(0.61, 0.62)
	Q2 vs Q5	0.75	(0.74, 0.75)	0.75	(0.74, 0.76)	0.74	(0.73, 0.75)
	Q3 vs Q5	0.83	(0.83, 0.84)	0.84	(0.83, 0.85)	0.82	(0.82, 0.83)
	Q4 vs Q5	0.91	(0.90, 0.91)	0.92	(0.91, 0.93)	0.90	(0.89, 0.91)
	Rural vs Q5	0.83	(0.82, 0.84)	0.84	(0.83, 0.85)	0.82	(0.81, 0.83)
Rostering status	Not rostered vs rostered	0.12	(0.12, 0.12)	0.11	(0.11, 0.11)	0.14	(0.14, 0.15)
	Virtually rostered vs rostered	0.63	(0.63, 0.63)	0.62	(0.62, 0.63)	0.64	(0.63, 0.64)

b. Multivariable logistic regression for early vs late stage of breast cancer diagnosis, adjusting for age, income quintile, rostering status, and region of birth							
Region of birth	Asia vs not an immigrant	1.00	(0.89, 1.13)	0.94	(0.81, 1.10)	1.08	(0.90, 1.30)
	Australia/North America vs Not an immigrant	1.26	(0.83, 1.91)	1.37	(0.77, 2.45)	1.15	(0.62, 2.11)
	Caribbean vs Not an immigrant	0.78	(0.59, 1.03)	0.69	(0.48, 0.98)	0.95	(0.60, 1.49)
	Europe/Former USSR vs Not an immigrant	0.95	(0.80, 1.14)	1.08	(0.84, 1.39)	0.84	(0.65, 1.08)
	North Africa/Middle East vs Not an immigrant	1.11	(0.86, 1.41)	0.96	(0.70, 1.32)	1.34	(0.90, 1.98)
	South America vs Not an immigrant	1.54	(1.00, 2.38)	1.31	(0.76, 2.25)	1.97	(0.95, 4.07)
	Sub-Saharan Africa vs Not an immigrant	0.63	(0.45, 0.89)	0.53	(0.34, 0.82)	0.81	(0.47, 1.39)
Time Period	Pandemic (April 1, 2020—March 31, 2022) vs Pre-pandemic (April 1, 2018—March 31, 2020)	0.99	(0.92, 1.05)				
Age		1.00	(1.00, 1.00)	1.00	(0.99, 1.00)	1.00	(1.00, 1.01)
Income quintile and rural status	Missing vs Q5	0.40	(0.20, 0.76)	0.50	(0.20, 1.24)	0.29	(0.11, 0.77)
	Q1 vs Q5	0.75	(0.67, 0.84)	0.75	(0.65, 0.87)	0.75	(0.64, 0.89)
	Q2 vs Q5	0.81	(0.72, 0.90)	0.82	(0.71, 0.95)	0.79	(0.67, 0.93)
	Q3 vs Q5	0.79	(0.71, 0.89)	0.81	(0.70, 0.93)	0.78	(0.66, 0.92)
	Q4 vs Q5	0.86	(0.77, 0.96)	0.89	(0.77, 1.03)	0.82	(0.70, 0.97)
	Rural vs Q5	0.98	(0.86, 1.11)	1.09	(0.91, 1.29)	0.87	(0.72, 1.05)
Rostering status	Not rostered vs Rostered	0.37	(0.32, 0.44)	0.36	(0.29, 0.45)	0.39	(0.31, 0.51)
	Virtually rostered vs Rostered	0.70	(0.64, 0.76)	0.69	(0.61, 0.78)	0.71	(0.62, 0.81)

**c.** Multivariable logistic regression for mastectomy vs breast-conserving surgery, adjusting for age, income quintile, rostering status, and region of birth							
Region of birth	Asia vs Not an immigrant	1.29	(1.18, 1.40)	1.31	(1.15, 1.48)	1.27	(1.13, 1.43)
	Australia/North America vs Not an immigrant	1.21	(0.91, 1.60)	1.29	(0.88, 1.89)	1.13	(0.75, 1.72)
	Caribbean vs Not an immigrant	0.80	(0.63, 1.02)	1.02	(0.74, 1.41)	0.63	(0.45, 0.89)
	Europe/Former USSR vs Not an immigrant	1.04	(0.91, 1.19)	1.07	(0.88, 1.30)	1.02	(0.84, 1.23)
	North Africa/Middle East vs Not an immigrant	0.99	(0.83, 1.18)	1.10	(0.85, 1.42)	0.89	(0.70, 1.14)
	South America vs Not an immigrant	0.73	(0.54, 0.98)	0.71	(0.46, 1.09)	0.75	(0.50, 1.13)
	Sub-Saharan Africa vs Not an immigrant	0.75	(0.55, 1.03)	0.74	(0.46, 1.20)	0.75	(0.49, 1.15)
Time Period	Pandemic (April 1, 2020—March 31, 2022) vs Pre-pandemic (April 1, 2018—March 31, 2020)	1.14	(1.08, 1.20)				
Age		0.99	(0.99, 0.99)	0.99	(0.99, 0.99)	0.99	(0.99, 0.99)
Income quintile and rural status	Missing vs Q5 (highest)	1.60	(0.89, 2.87)	1.15	(0.48, 2.77)	2.14	(0.96, 4.75)
	Q1 (lowest) vs Q5	1.13	(1.04, 1.22)	1.10	(0.98, 1.24)	1.15	(1.03, 1.29)
	Q2 vs Q5	1.11	(1.02, 1.20)	1.18	(1.05, 1.33)	1.04	(0.92, 1.16)
	Q3 vs Q5	1.02	(0.94, 1.11)	1.00	(0.89, 1.13)	1.04	(0.93, 1.16)
	Q4 vs Q5	1.10	(1.02, 1.19)	1.17	(1.04, 1.31)	1.04	(0.93, 1.16)
	Rural vs Q5	1.06	(0.97, 1.17)	1.17	(1.03, 1.34)	0.97	(0.85, 1.10)
Rostering status	Not rostered vs Rostered	1.07	(0.88, 1.31)	0.98	(0.74, 1.31)	1.15	(0.88, 1.51)
	Virtually rostered vs Rostered	1.12	(1.04, 1.20)	1.15	(1.03, 1.28)	1.09	(0.98, 1.21)

#### Diagnosis

For stage of diagnosis (Table 4b), there was no significant difference between the two time periods (AOR = 0.99 [95% CI (0.92–1.05)]). Similar patterns to screening were seen for income quintile and rostering status, whereby higher income and being rostered were associated with higher likelihood of early-stage diagnosis (rural residents were no longer significantly different than those who lived in the highest-income neighbourhoods). In the pre-pandemic period, women from the Caribbean and Sub-Saharan Africa had the lowest adjusted relative odds of early vs late-stage diagnosis (AOR = 0.69 [95% CI (0.48–0.98)] and AOR = 0.53 [95% CI (0.34–0.82)] respectively), although values were not significant for the pandemic period.

#### Surgery

For surgery (Table 4c), the adjusted relative odds of mastectomy vs breast-conserving surgery were higher in the pandemic period (AOR = 1.14 [95% CI (1.08–1.20)]). After adjustment, those in lower-income neighbourhoods trended toward higher likelihood of mastectomy vs breast-conserving surgery than those in the highest income quintile. Among immigrants, women from Asia had the highest relative odds of mastectomy in both time periods and overall (AOR = 1.29 [95% CI (1.18–1.40)]. In the pandemic period, women from the Caribbean stood out as being the only immigrant group with significantly lower relative odds of mastectomy: AOR = 0.63 [95% CI (0.45–0.89)].

## Discussion

In this provincial-level retrospective cohort study comparing a two-year pre-pandemic period to two years of the pandemic, we found that breast cancer screening rates in Ontario dropped from 59.4% to 51.0%, and that the absolute number of women diagnosed with breast cancer dropped from 18,821 to 14,269, in the pre-pandemic vs pandemic period. In multivariable regression analyses, screening significantly dropped (AOR = 0.69 [95% CI (0.69–0.69)]) from the pre-pandemic period, but there was no significant difference for stage of diagnosis (AOR = 0.99 [95% CI (0.92–1.05)]) between the two time periods, and the use of mastectomy vs breast-conserving surgery was higher in the pandemic period (AOR = 1.14 [95% CI (1.08–1.20)]). These findings are aligned with other Ontario studies that have found decreases in the proportion of people up to date on screening post-pandemic [6] and that have shown a higher proportion of breast cancer surgeries being mastectomies in the pandemic period [9]. Results for screening mammography are not surprising as cancer screening was paused for several months during the pandemic and only resumed at a gradual pace, leading to a backlog of more than 300,000 screening mammograms by December 2020 [22]. It is fortunate that this did not seem to result in an overall diagnostic stage shift in Ontario, although more subtle within-stage (tumour and nodal staging) shifts may have been missed. As postulated by Ko et al*.* [9], the relative increase in mastectomy may reflect prioritization of patients with higher-risk disease or patients choosing mastectomy over lumpectomy to minimize additional hospital visits for both potential additional surgery and/or radiation.

We also observed important differences for some subgroups, both overall, and within and across time periods. In particular, in our adjusted analyses, we found that women from the Caribbean had a higher likelihood of being up to date on screening regardless of time period than other groups including non-immigrants. Yet despite this screening advantage, women from the Caribbean still had lower odds of early-stage diagnosis in the pre-pandemic period. These results are consistent with other studies. Vahabi et al*.* found that women from the Caribbean and Latin America (grouped together as one region) had higher breast cancer screening rates than long-term residents of Ontario [14], while a 2019 study by the lead author found that Ontario women from Latin America and the Caribbean (grouped together as one region) were less likely to have a screen-detected breast cancer, and were diagnosed at a later stage than long-term residents of the province [16]. It is possible that the higher prevalence of aggressive triple-negative breast cancers (TNBC) among women of West African ancestry (which is the ancestry of most women from the Caribbean) may partly explain these findings [23, 24]. Notably, several studies have shown a high prevalence of TNBC in Caribbean countries [25–28]. TNBCs are the most aggressive subtype of breast cancer and consequently can develop between screening mammograms and are more likely to be diagnosed at a later stage [23, 29]. TNBCs also tend to occur in pre-menopausal women, before the Ontario age of average-risk screening eligibility of 50 years of age or older during our study time period [29].

Notably, we also found women from Sub-Saharan Africa were less likely to be diagnosed at an early stage in the pre-pandemic period; however, they had corresponding lower rates of screening uptake. Although cancer subtype may be one explanation, future qualitative work should explore the diagnostic experience for both Caribbean and Sub-Saharan African immigrant women to understand the role that systemic marginalization and racism may play in later stage of diagnosis. Ontario women from Latin America and the Caribbean have been found to have a longer median diagnostic interval after adjusting for stage [16], and in the US, Manik et al*.* found that Black women waited 1.75 times as long after an abnormal screening mammogram as white women to obtain a tissue diagnosis [30]. These results would not be explained by genetics but instead suggest that structural discrimination may be at play. Future qualitative work with women, clinicians and healthcare decision-makers should explore these findings with an equity lens, and should also explore why diagnostic stage differences abated for Caribbean women and why they had lower likelihood of mastectomy during the pandemic period. There may have been system or community-level changes that occurred during the pandemic that reduced inequities for this group, whether intentionally or not.

We also found that women from Europe and the former USSR had the lowest odds of screening relative to non-immigrants: OR = 0.62 [95% CI (0.61–0.63). The reasons for this cannot be ascertained from this study but our findings are aligned with work by Vahabi et al. who found the lowest screening uptake for women from South Asia, followed by women from Eastern Europe and Central Asia [14]. Notably**,** Eastern Europe and Central Asia also have higher breast cancer mortality rates and later stage at diagnosis compared with the rest of Europe [31]. Targeted screening initiatives in Ontario may be beneficial for this immigrant group.

Despite a universal healthcare system and regardless of time period, women living in lower-income neighbourhoods were significantly less likely to be screened, more likely to be diagnosed at stage III or IV, and more likely to have mastectomy than breast-conserving surgery than those in the highest income quintile. These findings are consistent with other Canadian studies which have shown income-related disparities in breast cancer screening [6], stage of diagnosis [32], post-mastectomy breast reconstruction [33], and mortality [32, 34]. In addition to these findings, the financial burden of cancer care in Canada is known to be high, and in a recent national study, Longo et al*.* reported that 41% of patients with cancer reported reducing their household spending, with this value rising to 52% for those earning less than $50,000 per year [35]. There continues to be an urgent need for clinicians and cancer care decision-makers to ensure that one’s income does not diminish the quality of cancer care one receives, from screening all the way through the cancer journey, and to identify and address the root causes of these income-related inequalities that still exist in a universal healthcare context.

This study has several limitations. First, as this is a secondary analysis of administrative data, we are currently unable to determine the causes for our findings. For example, we do not know if the pandemic itself was the reason for the changes observed, nor are we able to determine the reason for the relative increase in mastectomy. Second, the IRCC-PR database does not include immigrants who moved to another Canadian province before settling in Ontario or those who arrived in Canada before 1986. These groups would have been misclassified as long-term residents of the province. Third, we were unable to determine income at the individual level as these data are not available at ICES. Fourth, reliable data were not available to examine radiation treatment. However, the study is strengthened by being a population-level study with rich data in the context of a universal healthcare system.

## Conclusion

In our population-based study comparing a two-year pre-pandemic period to two years of the pandemic, we found that screening significantly dropped (AOR = 0.69 [95% CI (0.69–0.69)]), there was no significant difference for stage of diagnosis (AOR = 0.99 [95% CI (0.92–1.05)]), and the use of mastectomy vs breast-conserving surgery increased (AOR = 1.14 [95% CI (1.08–1.20)]). We also found that women from the Caribbean had lower odds of early-stage diagnosis in the pre-pandemic period despite an apparent screening advantage. Future work should further explore the reasons for these findings and potential system-level solutions.

## Data Availability

The dataset from this study is held securely in coded form at ICES. While legal data sharing agreements between ICES and data providers (e.g., healthcare organizations and government) prohibit ICES from making the dataset publicly available, access may be granted to those who meet pre-specified criteria for confidential access, available at www.ices.on.ca/DAS (email: das@ices.on.ca). The full dataset creation plan and underlying analytic code are available from the authors upon request, understanding that the computer programs may rely upon coding templates or macros that are unique to ICES and are therefore either inaccessible or may require modification.

## References

[1] Panchuk J et al (2024) COVID-19 in a rural intensive care unit in Northern British Columbia: descriptive analysis of outcomes and demands on rural resources. Can J Rural Med 29(3):109–116 39155633 10.4103/cjrm.cjrm_42_23

[2] Douglas IS, Mehta A, Mansoori J (2024) Policy proposals for mitigating ICU strain: insights from the COVID-19 pandemic. Ann Am Thorac Soc 21(12):1633–1642 39236274 10.1513/AnnalsATS.202404-356FR PMC11622822

[3] Winkelmann J et al (2022) European countries’ responses in ensuring sufficient physical infrastructure and workforce capacity during the first COVID-19 wave. Health Policy 126(5):362–372 34311982 10.1016/j.healthpol.2021.06.015 PMC9187509

[4] Ahmed S (2022) Cancer care during the COVID-19 pandemic: challenges and adaptations. Curr Oncol 30(1):45–47 36661653 10.3390/curroncol30010004 PMC9857587

[5] Malagón T et al (2022) Predicted long-term impact of COVID-19 pandemic-related care delays on cancer mortality in Canada. Int J Cancer 150(8):1244–1254 34843106 10.1002/ijc.33884 PMC9015510

[6] Lofters A et al (2023) Cancer screening disparities before and after the COVID-19 pandemic: a population-based study in Ontario Canada. JAMA Network Open 6(11):e2343796 37983033 10.1001/jamanetworkopen.2023.43796 PMC10660460

[7] Walker MJ et al (2022) Delivery of cancer care in Ontario, Canada, during the first year of the COVID-19 pandemic. JAMA Netw Open 5(4):e228855 35467731 10.1001/jamanetworkopen.2022.8855 PMC9039771

[8] Roberson ML et al (2022) Trends in surgical treatment of early-stage breast cancer reveal decreasing mastectomy use between 2003 and 2016 by age, race, and rurality. Breast Cancer Res Treat 193(2):445–454 35286524 10.1007/s10549-022-06564-w PMC9098687

[9] Ko G et al (2024) Impact of the COVID-19 pandemic on breast cancer surgeries in a Canadian population. Breast Cancer Res Treat. 10.1007/s10549-024-07547-9 39543062 10.1007/s10549-024-07547-9 PMC11787185

[10] Roskam JS et al (2023) The effects of the COVID-19 pandemic on mastectomy outcomes for breast cancer. Clin Breast Cancer 23(4):431–435 36990842 10.1016/j.clbc.2023.02.010 PMC9951028

[11] Fujita M et al (2023) Impact of coronavirus disease 2019 pandemic on breast cancer surgery using the National Database of Japan. Sci Rep 13(1):4977 36973536 10.1038/s41598-023-32317-w PMC10041497

[12] Xia Y et al (2022) Geographic concentration of SARS-CoV-2 cases by social determinants of health in metropolitan areas in Canada: a cross-sectional study. CMAJ 194(6):E195–E204 35165131 10.1503/cmaj.211249 PMC8900797

[13] Vahabi M et al (2017) Breast cancer screening utilization among women from Muslim majority countries in Ontario, Canada. Prev Med 105:176–183 28916289 10.1016/j.ypmed.2017.09.008

[14] Vahabi M et al (2016) Breast cancer screening disparities among immigrant women by world region of origin: a population-based study in Ontario. Canada. Cancer Med 5(7):1670–86 27105926 10.1002/cam4.700 PMC4944895

[15] Vahabi M et al (2015) Breast cancer screening disparities among urban immigrants: a population-based study in Ontario, Canada. BMC Public Health 15:679 26194189 10.1186/s12889-015-2050-5 PMC4508905

[16] Lofters AK et al (2019) Disparities in breast cancer diagnosis for immigrant women in Ontario and BC: results from the canIMPACT study. BMC Cancer 19(1):42 30626375 10.1186/s12885-018-5201-0 PMC6327524

[17] Ginsburg OM et al (2015) A population-based study of ethnicity and breast cancer stage at diagnosis in Ontario. Curr Oncol 22(2):97–104 25908908 10.3747/co.22.2359 PMC4399617

[18] Kumachev A, Trudeau ME, Chan KK (2016) Associations among socioeconomic status, patterns of care and outcomes in breast cancer patients in a universal health care system: Ontario’s experience. Cancer 122(6):893–898 26696022 10.1002/cncr.29838

[19] Census Profile, 2021 Census of Population - Ontario. (2023). Available from: https://www12.statcan.gc.ca/census-recensement/2021/dp-pd/prof/details/page.cfm?Lang=E&SearchText=Ontario&DGUIDlist=2021A000235&GENDERlist=1,2,3&STATISTIClist=1,4&HEADERlist=0.

[20] Screening Programs - Cancer Care Ontario. (2022). Available from: https://www.cancercareontario.ca/en/cancer-care-ontario/programs/screening-programs.

[21] Austin PC (2009) Using the standardized difference to compare the prevalence of a binary variable between two groups in observational research. Commun Stat Simul Comput 38:1228–1234

[22] Walker MJ et al (2021) Measuring the impact of the COVID-19 pandemic on organized cancer screening and diagnostic follow-up care in Ontario, Canada: a provincial, population-based study. Prev Med 151:106586 34217413 10.1016/j.ypmed.2021.106586 PMC9755643

[23] Gupta GK et al (2020) Perspectives on triple-negative breast cancer: current treatment strategies, unmet needs, and potential targets for future therapies. Cancers (Basel) 12(9):2392 32846967 10.3390/cancers12092392 PMC7565566

[24] Newman LA (2017) Breast cancer disparities: socioeconomic factors versus biology. Ann Surg Oncol. 10.1245/s10434-017-5977-1 28766222 10.1245/s10434-017-5977-1

[25] Hercules SM et al (2020) High triple-negative breast cancer prevalence and aggressive prognostic factors in Barbadian women with breast cancer. Cancer 126(10):2217–2224 32154924 10.1002/cncr.32771

[26] George SHL et al (2021) Gene sequencing for pathogenic variants among adults with breast and ovarian cancer in the Caribbean. JAMA Netw Open 4(3):e210307 33646313 10.1001/jamanetworkopen.2021.0307 PMC7921902

[27] Samaroo K et al (2021) Breast cancer in the Caribbean. Cureus 13(8):e17042 34522520 10.7759/cureus.17042 PMC8428164

[28] Copeland J et al (2021) Breast cancer in Jamaica: stage, grade and molecular subtype distributions across age blocks, the implications for screening and treatment. World J Oncol 12(4):93–103 34349853 10.14740/wjon1389 PMC8297049

[29] Chacón RD, Costanzo MV (2010) Triple-negative breast cancer. Breast Cancer Res 12 Suppl 2(Suppl 2):S3 21050424 10.1186/bcr2574 PMC2972557

[30] Manik R et al (2024) Racial disparities and strategies for improving equity in diagnostic follow-up for abnormal screening mammograms. JCO Oncol Pract 20(10):1367–1375 38900977 10.1200/OP.23.00782 PMC11473231

[31] Znaor A et al (2023) Breast and cervical cancer screening practices in nine countries of Eastern Europe and Central Asia: a population-based survey. J Cancer Policy 38:100436 37544479 10.1016/j.jcpo.2023.100436 PMC10695765

[32] Ruan Y et al (2024) The association between neighborhood-level income and cancer stage at diagnosis and survival in Alberta. Cancer 130(4):563–575 37994148 10.1002/cncr.35098

[33] Huang Y et al (2024) Geographic variation in breast reconstruction surgery after mastectomy for females with breast cancer in Alberta, Canada. Can J Surg 67(2):E172–E182 38670581 10.1503/cjs.003823 PMC11052580

[34] Nasiri N, Hu M, Hajizadeh M (2024) Trends in socioeconomic inequalities in breast cancer mortality in Canada: 1992–2019. Breast Cancer Res Treat 205(3):533–543 38502420 10.1007/s10549-024-07277-y

[35] Longo CJ et al (2024) Patient and family financial burden in cancer: a focus on differences across four provinces, and reduced spending including decisions to forego care in Canada. Curr Oncol 31(5):2713–2726 38785487 10.3390/curroncol31050206 PMC11119025

